# Bone mesenchymal stem cells ameliorate ischemia/reperfusion-induced damage in renal epithelial cells via microRNA-223

**DOI:** 10.1186/s13287-017-0599-x

**Published:** 2017-06-15

**Authors:** Xiaopeng Yuan, Xiaoping Wang, Chuanbao Chen, Jian Zhou, Ming Han

**Affiliations:** Third Division of Organ Transplant Center, Eastern Campus of First Affiliated Hospital, Sun Yat-sen University, Guangzhou, 510700 People’s Republic of China

**Keywords:** Ischemia/reperfusion, Mesenchymal stem cell, microRNA-223, NLR family-pyrin domain containing 3, Inflammation

## Abstract

**Background:**

Recent studies have indicated that microRNA-223 (miR-223) plays a role in the tissue-protective effect of mesenchymal stem cells (MSCs). NLR family-pyrin domain containing 3 (NLRP3) was reported to affect a renal ischemia/reperfusion (I/R) injury by exerting a direct effect on the renal tubular epithelium. Therefore, we investigated how miR-223 and NLRP3 might function in kidneys exposed to conditions of ischemia and subsequent reperfusion.

**Methods:**

Hypoxia/reoxygenation (H/R) murine renal tubular epithelial cells (RTECs) were cocultured with either MSCs or hypoxia-pretreated MSCs (htMSCs), after which the RTECs were examined for their viability and evidence of apoptosis. Next, miR-223 expression in the MSCs was downregulated to verify that MSCs protected RTECs via the transport of miR-223. Kidney I/R KM/NIH mouse models were created and used for in vivo studies.

**Results:**

The results showed that coculture with MSCs significantly increased the viability of RTECs and decreased their rates of apoptosis. The levels of hepatocyte growth factor (HGF), insulin-like growth factor-1 (IGF-1), transforming growth factor beta (TGF-β), and vascular endothelial growth factor (VEGF) in samples of coculture supernatants were higher than those in samples of non-coculture supernatants. A bioinformatics analysis revealed a targeting relationship between miR-223 and NLRP3. A dual luciferase assay showed that miR-223 inhibited NLRP3 expression. The htMSCs displayed a protective function associated with an upregulation of miR-223 as induced by Notch1 and the downregulation of NLRP3. Conversely, inhibition of miR-223 impeded the protective effect of MSCs. In the I/R mouse models, injection of either MSCs or htMSCs ameliorated the damage to kidney tissue, while suppression of miR-223 expression in MSCs reduced their protective effect on mouse kidneys.

**Conclusions:**

Our results demonstrate that miR-223 and NLRP3 play important roles in the treatment of renal tissue injuries with transplanted MSCs.

## Background

Renal blood flow is always temporarily disrupted during various surgical treatments, including those performed for the purposes of kidney transplantation, partial nephrectomy, aortic bypass, and treatment of sepsis [1–4]. Although the re-initiation of blood flow to ischemic kidney regions restores their function, the restoration process itself can further damage kidney tissue, resulting in an ischemia/reperfusion (I/R) injury [5]. Patients who are hospitalized due to acute renal failure caused by an I/R injury have high rates of morbidity and mortality. In recent years, considerable effort has being made to improve the treatment of renal I/R injuries, and a variety of agents and growth factors have been proven effective for treating these disorders. However, most of these treatment modalities were validated in animal models, and no significant efficacy was demonstrated in human clinical trials [6]. Therefore, the development of novel and effective strategies for managing and treating renal I/R injuries remains a high priority.

In recent years, several studies have demonstrated that mesenchymal stem cells (MSCs) may help attenuate or prevent an I/R injury in multiple organs, including the kidney, and may exert that effect via paracrine/autocrine mechanisms or transdifferentiation into local cell types [7–10]. MSCs are multipotent cells capable of differentiating into various types of specialized mesenchymal cells such as osteoblasts, chondrocytes, adipocytes, and tenocytes [11]. MSCs are easier to culture than embryonic stem cells (ESCs) and other adult stem cells [8], and their functional properties make them promising candidates for use in tissue engineering studies and as agents for treating various diseases, including renal I/R injuries [11]. Semedo et al. [12] showed that MSCs could modulate I/R injuries in renal tissue by inhibiting inflammatory responses. Rosova et al. [13] proposed that the beneficial effects of MSCs could be primarily attributed to their ability to mediate complex paracrine actions. Moreover, MSCs were shown to improve the recovery of I/R-injured rodents even if they were infused starting at 24 h after the injury, which suggests that MSCs actively participate in repairing I/R-injured tissues [13–15].

The mechanism by which MSCs protect kidney tissue is complicated, and may involve the paracrine/autocrine effects of cytokines, their transdifferentiation into the local cell type, and/or immunomodulation [8, 16]. A previous study showed that administration of MSCs into a DCD rat kidney alleviated kidney tissue damage [17]. Moreover, recent evidence suggests that the NLR family-pyrin domain containing 3 (NLRP3) inflammasome may upregulate cytokine production and play a critical role in renal injuries [18]. NLRP3 may also contribute to renal I/R injuries by directly affecting the renal tubular epithelium [19]. Given that NLRP3 is negatively regulated by microRNA-223 (miR-223) [20], an enhanced expression of miR-223 might be expected to ameliorate the damage to renal tissues. The cardioprotective effect of MSCs is mediated through exosomal miR-223. Moreover, Notch receptors expressed in MSCs were proven to be upstream regulators of miR-223 [21, 22]. Therefore, we hypothesized that MSCs might exert their protective function by suppressing NLRP3 via Notch-induced activation of miR-223. We then tested our hypothesis in a series of in vitro and in vivo assays.

The effect of MSC administration and the role of miR-223/NLRP3 signaling in renal I/R injuries were examined using hypoxia/reoxygenation (H/R) murine renal tubular epithelial cells (RTECs), and were then verified in a renal I/R KM/NIH mouse model. Our results not only verified the protective effect of MSCs on kidney tissue, but helped explain the mechanism by which MSCs limit the extent of I/R injuries.

## Methods

### Preparation of cells

Murine RTECs were purchased from Zhongqiaoxinzhou Cell Research (Cat. No. M4100; Shanghai, China) and cultured in Dulbecco’s modified Eagle’s medium (DMEM) supplemented with 10% fetal bovine serum (FBS) and penicillin/streptomycin at 37 °C in a 5% CO_2_ atmosphere. MSCs were generated from bone marrow tissue extracted from the femurs of sacrificed mice, and suspended in sterile phosphate-buffered saline (PBS). The extracted cells were then resuspended in DMEM, filtered through 70-μm mesh lattice, and plated in flasks containing DMEM supplemented with 10% FBS (Thermo Fisher Scientific, Waltham, MA, USA). After 72 h of culture, any nonadherent cells were removed, and the adherent cells were passed at a low density into new flasks. Cells that displayed a typical spindle-shaped appearance were used for subsequent assays.

### Induction of the H/R RTEC model and coculture

To detect the effect of MSCs on miR-223/NLRP3 activity, the RTECs were divided into the following four groups: 1) RTEC group (normal RTECs); 2) HR RTEC group, (H/R-treated RTECs); 3) HR RTEC + MSC group (H/R-treated RTECs cocultured with MSCs for 48 h); and 4) HR RTEC + htMSC group (H/R-treated RTECs cocultured with hypoxia-pretreated MSCs (htMSCs) for 48 h).

To detect the role of the miR-223/NLRP3 pathway in renal I/R injuries, the RTECs were divided into the following two groups: 1) Negative control group (H/R-treated RTECs cocultured with MSCs transfected with negative control inhibitors); and 2) miR-223 inhibitor group (H/R-treated RTECs cocultured with MSCs transfected with a miR-223-specific inhibitor).

For the H/R treatment, RTECs were plated into six-well plates and exposed to H/R conditions as previously described [23]. Briefly, the RTECs were inoculated at concentrations of 5 × 10^5^ cells per well and incubated in DMEM culture medium containing 10% FBS. To synchronize cell growth, the culture medium waschanged to serum-free DMEM at 24 h before treatment. Next, the ischemia/hypoxia medium (glucose; 10,000 mg/L) was added to the cells, and the plates were put into double-layered, sealed, self-styled bags with an anaerobic indicator. The bags were then filled with low-oxygen gas (5% CO_2,_ 95% N_2_) to expel the air. When the purple anaerobic indicator turned red, the bag was sealed to maintain the anaerobic condition for 2 h, after which oxygen was added to the bag and the medium was changed to DMEM with FBS for further incubation. Hypoxia treatment has been reported to improve the therapeutic efficacy of MSCs [13]. For hypoxia pretreatment of MSCs, the cells were cultured in 12-well plates with DMEM supplemented with 10% FBS, 50 μg/mL penicillin, and 50 mg/L gentamicin (Sigma, St. Louis, MO, USA) prior to being cocultured with RTECs. Each parameter examined in the following assays was measured in at least three identical samples. A double-chamber coculture system was used for cell coculture. The system was separated into upper and lower chambers by a 0.4-μm pore-size membrane (12-well insert; BD Biosciences. Franklin Lakes, NJ, USA). RTECs (1 × 10^5^ cells/well) were cultured in the lower chamber and MSCs or htMSCs (1 × 10^5^ cells/well) were cultured in the upper chamber. Cells were harvested after 24 h, 48 h, and 72 h of culture, respectively.

### Creation of renal I/R mouse models and transplantation of MSCs

Twenty-four KM/NIH mice (female, 34 ± 2.1 g) were used for in vivo assays and were randomly assigned to four different groups (six mice per group): 1) Control group (mice underwent I/R treatment and were abdominally intravenously injected with the same volume of normal saline as used for negative control groups); 2) MSC group (15 min before I/R treatment the mice were abdominally intravenously injected with 2 × 10^6^ MSCs in a volume of 200 μL and then transfected with a negative control inhibitor); 3) miR-223 inhibitor group (15 min before I/R treatment the mice were abdominally intravenously injected with 2 × 10^6^ miR-223 knockdown MSCs); and 4) htMSC group (15 min before I/R treatment the mice were abdominally intravenously injected with 2 × 10^6^ hypoxia-pretreated MSCs). For induction of the renal I/R model, mice were anesthetized with isoflurane and their rectal temperature was maintained at 37 °C. After a midabdominal laparotomy, the kidneys were exposed and the renal pedicles were clamped with atraumatic vascular clamps for 60 min. While the clamps were applied, the left carotid artery was cannulated with PE-50 tubing to allow for intraaortic cell delivery immediately after blood flow was reestablished. All mice were sacrificed at 24 h after their operation.

### Transfections of MSCs

MSCs (1 × 10^5^) were seeded into six-well plates (18 replications). Transfection or hypoxic stimulation was performed until the cells reached 70–80% confluence. A miR-223-specific inhibitor (sequence: 5′-ACAGUCAAACAGUUUAUGGGUU-3′) or negative control (sequence: 5′-UGCGCUGCUGGUGCCAACCCUAUUCU-3′) purchased from Jikai Biosciences (Shanghai, China) was transfected into the MSCs using Lipofectamine 2000 reagent (Thermo Fisher Scientific Waltham, MA, USA) according to the manufacturer’s instructions. At 24 h posttransfection, the MSCs were harvested and used for coculture with RTECs. The knockdown effect in the MSCs lasted for 96 h.

### Dual luciferase assay

Full length NLRP3 mRNA (mNLRP3) 3′-untranslated region (UTR) was inserted into the psiCHECK-2 luciferase reporter vector to construct psiCHECK-2-mNLRP3-wt. The mutated form of mNLRP3 3′-UTR was used to construct psiCHECK-2-mNLRP3-mut. ViaFect™ Transfection Reagent (Promega, Fitchburg, WI, USA) was used to cotransfect 1 μg of psiCHECK-2-mNLRP3-wt or psiCHECK-2-mNLRP3-mut with 50 nm/L miR-223 mimic or miR-223 inhibitor into RTECs. Dual luciferase activity was assayed 48 h later.

### CCK-8 assay

The cell counting kit-8 (CCK-8) assay was used to detect RTEC proliferation in the different groups. Briefly, RTECs in the coculture system were harvested at 24 h, 48 h, and 72 h, respectively, after which 100 μL of CCK8 solution (Dojindo Molecular Technologies, Gaithersburg, MD, USA) was added to each well. After 4 h of incubation at 37 °C the optical density of each well at 450 nm was read with a microplate reader (Thermo Plate, Rayto Life and Analytical Science C. Ltd., Shenzhen, Guangdong, China).

### Flow cytometry assay

The apoptotic rates of RTECs sampled at 48 h in the different groups were determined by flow cytometry. Cells in different groups were collected after centrifugation at 600 g for 5 min, and their apoptotic rates were measured using an Annexin V-FITC Apoptosis Detection Kit (WLA001c, Wanleibio, Shenyang, China) according to instructions provided by the manufacturer. Briefly, 5 μL of Annexin V was added to different wells containing RTECs. After a 10-min incubation with Annexin V at room temperature, the cells were resuspended in 1 × binding buffer, and 5 μL of propidium iodide was added to each sample. Next, the apoptotic rates were analyzed by flow cytometry (Accuri C6, BD, San Jose, CA, USA). The overall apoptotic rate (UR + LR—all apoptosis cell percentage) was equal to the sum of the late apoptotic rate (UR, upper right quadrant—advanced stage apoptosis cell percentage) and the early apoptotic rate (LR, lower right quadrant—prophase apoptosis cell percentage).

### Enzyme-linked immunosorbent assay

The levels of renal protecting cytokines, including hepatocyte growth factor (HGF; Cat. No. E-EL-R0496c, Elabscience-Biotech Co. Ltd., Wuhan, China), transforming growth factor beta (TGF-β; Cat. No. CSB-E04727r, Cusabio Biotech, Wuhan, China), insulin-like growth factor-1 (IGF-1; Cat. No. CSB-E04582r, Cusabio Biotech, Wuhan, China), and vascular endothelial growth factor (VEGF; Cat. No. CSB-E04757r, Cusabio Biotech, Wuhan, China) were measured using enzyme-linked immunosorbent assay (ELISA) kits according to the manufacturers’ instructions. Serum blood urea nitrogen (BUN) levels were detected using an ELISA kit manufactured by Bio-Medical Assay, Beijing, China.

### Quantitative real-time polymerase chain reaction (qRT-PCR)

The total RNA in different cell samples at 48 h was extracted using an RNA Purified Total RNA Extraction Kit according to the manufacturer’s instructions (Cat. No. RP1201, BioTeke, Beijing, China). *β-actin* and *U6* were used as internal reference genes. Next, Super M-MLV reverse transcriptase (Cat. No. RP6502, BioTeke, Beijing, China) was used to reverse transcribe samples of total RNA into cDNA templates. Each 20 μL RT-PCR reaction mixture consisted of 10 μL Bestar® SybrGreen qPCR Master Mix, 0.5 μL of each primer (miR-223, forward: 5′-ACACTCCAGCTGGGTGTCAGTTTGTCAAATAC-3′, universally reverse: 5′-CTCAACTGGTGTCGTGGA-3′; NLRP3, forward: 5′-CAGAAGGCTGTGAGGGGAGA-3′, reverse: 5′-GCAGACCAGGGGGATGAAG-3′; Notch1, forward: 5′-GAGATTGGCTCCTATCGCTG-3′, reverse: 5′-GGGCAGTCATCCACATTTTC-3′; Bcl-2, forward: 5′-GGCATCTTCTCCTTCCAGC-3′, reverse: 5′-CCTCCCCCAGTTCACCC-3′; Bcl-XL, forward: 5′-TCGCCAGCCTCTCTCAGC-3′, reverse: 5′-AGACCCCCAGTGCCATCA-3′; Caspase-1, forward: 5′-CCTCAAGTTTTGCCCTTTAGA-3′, reverse: 5′-TACGAGTGGGTGTTTTCATTATT-3′; Caspase-3, forward: 5′-GGGTGCGGTAGAGTAAGCA-3′, reverse: 5′-GGAACGAACGGACCTGTG-3′; β-actin forward: 5′-GGAGATTACTGCCCTGGCTCCTA-3′, reverse: 5′-GACTCATCGTACTCCTGCTTGCTG-3′; U6 forward: 5′-CTCGCTTCGGCAGCACA-3′, reverse: 5′-AACGCTTCACGAATTTGCGT-3′), 2 μL cDNA template, and 7 μL Rnase-free H_2_O. The amplification parameters were as follows: denaturation at 95 °C for 2 min, followed by 40 cycles at 94 °C for 20 s, 58 °C for 20 s, and 72 °C for 30 s, after which the reaction was stopped by lowering the temperature to 4 °C for 5 min. Relative expression levels of the targeted molecules were calculated by an Exicycler™ 96 PCR system (Bioneer, Alameda, CA, USA) using the 2^–△△ct^ method.

### Western blot assays

The total proteins in different groups were extracted using a total protein extraction kit according to the manufacturer’s instructions (Cal. No. WLA019, Wanleibio, China). GAPDH was used as an internal reference. The protein concentration in each sample was determined using the BCA method. A 20-μL aliquot of protein (40 μg) was separated by electrophoresis on a 10% sodium dodecylsulfate polyacrylamide gel. Following separation, the targeted proteins were transferred onto polyvinylidene difluoride (PVDF; BD, San Jose, CA, USA) sheets, which were then washed with TTBS for 5 min prior to being incubated in a powdered skim milk solution for 1 h. Primary antibodies against NLRP3 (1:400, Santa Cruz Biotechnology, Santa Cruz, CA, USA), Notch1 (1:1500, Santa Cruz Biotechnology Santa Cruz, CA, USA), Bcl-2 (1:500, Santa Cruz Biotechnology, Santa Cruz, CA, USA), Bcl-XL (1:1000, Santa Cruz Biotechnology, Santa Cruz, CA, USA), caspase-1 (1:2000, Abcam, Cambridge, MA, USA), caspase-3 (1:800, Abcam, Cambridge, MA, USA), and glyceraldehyde-3-phosphate dehydrogenase (GAPDH; 1:4000, Abcam; Cambridge, MA, USA) were incubated with the membranes at 4 °C overnight. Following incubation, the membranes were washed four times with TTBS, after which secondary HRP goat anti-rabbit antibodies (1:20,000, Cat. No. BA1054, Wuhan, China) were added to the mixture and incubated with the membranes for 45 min at 37 °C. After an additional six washes with TTBS, the blots were developed using Beyo ECL Plus reagent, and the results were detected with a gel imaging system. The relative levels of BDNF expression in the different samples were calculated using Gel-Pro-Analyzer (Media Cybernetics, Rockville, MD, USA).

### Immunofluorescence assays

RTECs which had received the different respective treatments for 48 h were seeded into 14-well chambers, washed with PBS, and fixed with 4% paraformaldehyde for 15 min. The cells were then permeabilized with 0.5% Triton X-100 for 30 min. Next, the cells were washed three times with PBS (5 min per wash), and blocked with 10% goat serum for 15 min. Primary rabbit polyclonal antibodies to Notch1 (1:200, Santa Cruz Biotechnology, USA) and NLRP3 (1:100, Santa Cruz Biotechnology, USA) were then added, and the cells were incubated overnight at 4 °C in 1% goat serum. The cells were then incubated and stained with a fluorescein isothiocyanate secondary antibody (goat-anti-rabbit-Alexa 594 conjugated antibodies, Life Technologies, USA) for 1 h. Following incubation with the secondary antibody, the cells were washed and then stained with 4,6-diamino-2-phenyl indole (DAPI; Life Technologies, USA) for 5 min at room temperature. After three 5-min washes with PBS buffer, the slides were fixed and imaged with a fluorescence microscope at × 100 magnification.

### Hematoxylin and eosin (H&E) staining

The histological changes in sections of kidney tissue from the different groups were observed using H&E staining. Briefly, the tissues were placed into Bouin’s solution (4% formaldehyde) for perfusion fixation, after which they were dehydrated using different concentrations of alcohol and vitrified in dimethylbenzene. The tissues samples were then embedded in paraffin, sectioned, stained with H&E, and examined under a microscope at × 200 magnification. Following H&E staining, the cell nuclei were stained blue and cytoplasmic areas were stained red.

### Periodic acid-silver methenamine (PASM) staining

PASM staining was used as an optimal method for identifying the extracellular matrix in sections of renal tissue. Briefly, tissues sections were stained in 1% acid periodic, after which they were treated with an Ag-methenamine solution and stained with AuCl. After staining, collagen fibers appeared as dark strands against a pink background.

### Terminal deoxynucleoitidyl transferase-mediated nick-end labeling (TUNEL) staining

The rates of cellular apoptosis in samples of renal tissue were determined using TUNEL staining. Briefly, tissue sections were permeabilized with 50 μL 0.1% Triton X-100 at room temperature for 8 min. Next, the sections were washed three times with PBS buffer (5 min per wash) and then incubated in 3% H_2_O_2_ for 10 min at room temperature. After another three 5-min washes in PBS buffer, the sections were covered with TUNEL reaction solution and incubated at 37 °C for 1 h in a darkened humidified chamber. Following incubation, the sections were washed three times with PBS buffer (5 min per wash), and then incubated with Converter-POD at 37 °C for 30 min. The reaction was then stopped, and the cells were restained with hematoxylin. The stained renal tissues were then examined under a microscope (CX41, Olympus, Shinjuku, Tokyo, Japan) at × 200 magnification.

### Immunohistochemical detection methods

Sections prepared from different tissue samples were examined independently by senior pathologists, and then immediately frozen for Notch1 detection. Tissue slides to be used for immunohistochemical assays were stored at 60 °C overnight, and then incubated with dimethylbenzene for dewaxing. The slides were then hydrated with different concentrations of alcohol (100% for 5 min, 95% for 5 min, 85% for 5 min, and 75% for 5 min) and washed with H_2_O_2_ for 5 min. Slides with tissue sections from different animals were fixed using a methanol solution containing 3% H_2_O_2_, and then blocked with 1% bovine serum albumin (BSA) for 30 min at 37 °C. They were then incubated at 37 °C for 30 min with a primary antibody against Notch1 (1:200, Abcam, Cambridge, MA, USA), before being incubated at 4 °C overnight. After four cycles of washing in 0.01 M PBS (5 min per cycle), a secondary antibody (goat-anti-rabbit-Alexa 594 conjugated antibody; Thermo Fisher Scientific, Waltham, MA, USA) was added to the slides, which were then incubated at 37 °C for 30 min. After another four cycles of washing in PBS, DAB was added to the slides and reacted for 3–10 min until the reaction was stopped by addition of ddH_2_O. The slides were then restained with hematoxylin, dehydrated, and examined under a microscope at × 200 magnification.

### Creatinine clearance rate test

Urine was collected within 24 h after each operation and the creatinine levels in plasma and urine samples were detected with a creatinine assay kit (BioAssay Systems, Hayward, CA, USA). The creatinine clearance rate (Ccr) was calculated according to the Cockcroft-Gault formula: (urinary volume (mL, 24 h) × urinary creatinine (μmol/L))/(serum creatinine (μmol/L) × urine collection length (min)).

### Statistical analysis

All the data are expressed as means ± SD. One-way analysis of variance (ANOVA), ANOVA for repeated measurements, and Duncan’s post-hoc test were all performed using a general linear model. A *P* value < 0.05 indicated statistical significance. All the statistical analyses were performed using SPSS Statistics for Windows, version 19.0 (IBM, Armonk, NY, USA).

## Results

### Coculture of RTECs with MSCs increased the viability of RTECs and suppressed their rates of apoptosis

**Fig. 1:**
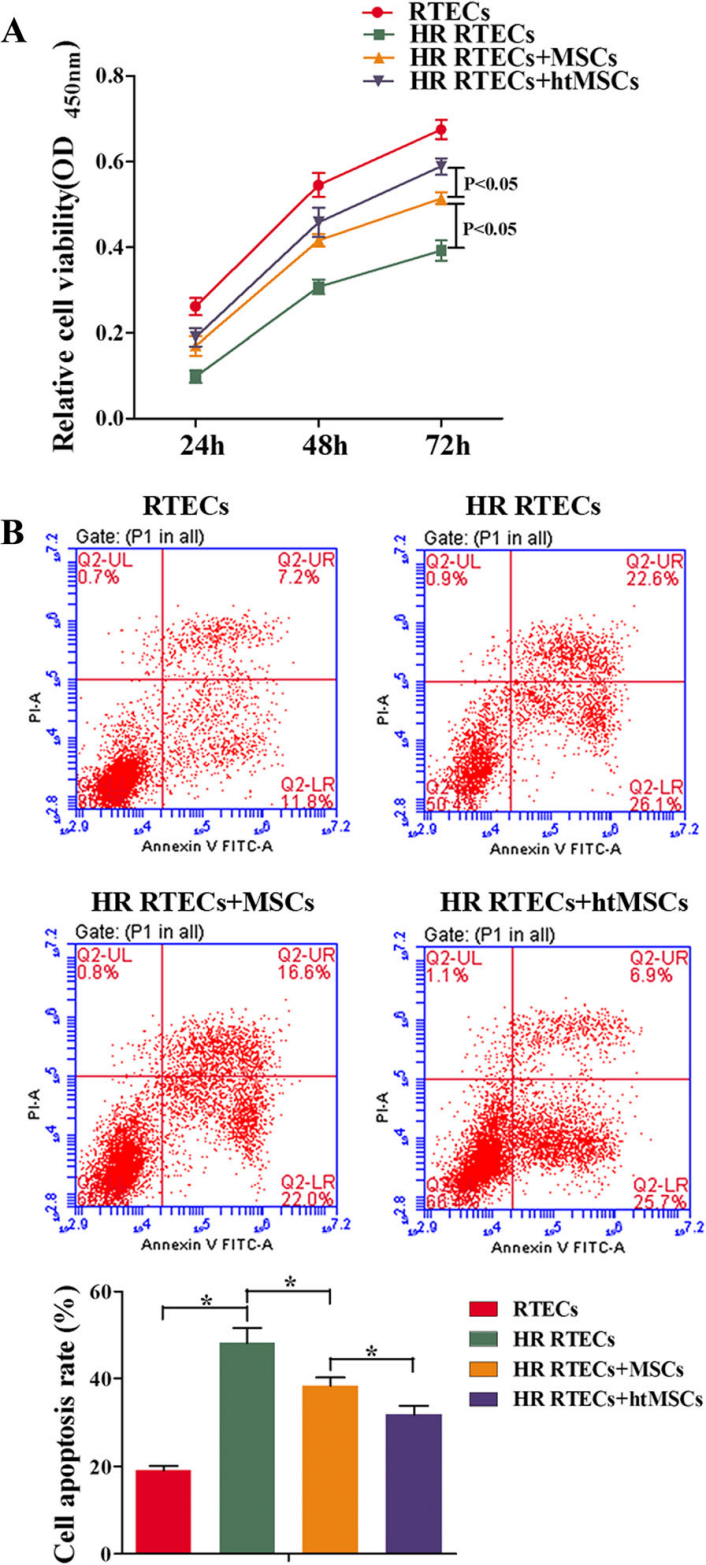
Coculture with mesenchymal stem cells (*MSCs*) increased cell viability and suppressed apoptosis of renal tubular epithelial cells (*RTECs*). RTECs were treated with hypoxia/reoxygenation (*HR*) stimulation and then cocultured with MSCs or hypoxia-pretreated MSCs (*htMSCs*) in a double-chamber. RTECs were cultured in the lower chamber and MSCs or htMSCs were cultured in the upper chamber. **a** RTEC viability was measured with the CCK-8 assay. **b** Representative images of apoptotic RTECs at 48 h as detected by flow cytometry. *P < 0.05, *n* = 3. *OD* optical density, *PI* propidium iodide

H/R treatment of RTECs decreased their viability and enhanced their rates of apoptosis (Fig. 1), while coculture with MSCs attenuated the effects of H/R. When examined at 72 h, the difference in optical density (OD_450_) values between H/R RTECs that were cocultured with MSCs and H/R RTECs that were not cocultured with MSCs was statistically significant (*P* < 0.05; Fig. 1a). Similar differences were detected in the apoptosis rates of the two groups of cells (Fig. 1b). Moreover, the cellular activity of RTECs treated with htMSCs at 72 h was higher than that of RTECs treated with normal MSCs. This suggested that coculture with MSCs can protect RTECs from H/R-induced injuries, and hypoxia pretreatment of MSCs enhanced their protective effect.

### Expression of miR-223 was upregulated in RTECs cocultured with MSCs and htMSCs

**Fig. 2:**
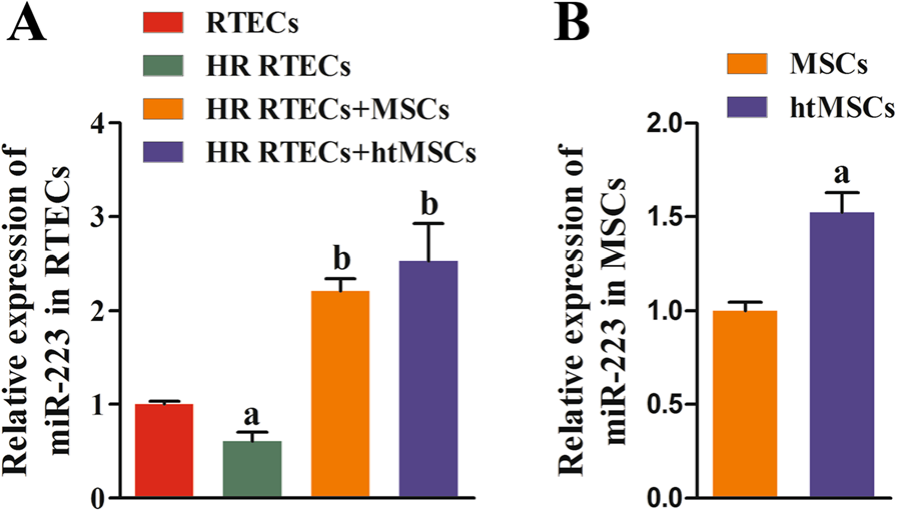
Expression of miR-223 was upregulated in renal tubular epithelial cells (*RTECs*) cocultured with mesenchymal stem cells (*MSCs*) or hypoxia-pretreated MSCs (*htMSCs*). RTECs were treated with hypoxia/reoxygenation (*HR*) stimulation and then cocultured with MSCs or htMSCs in a double-chamber. Cells were harvested at 48 h. **a** Expression of miR-223 in RTECs was detected by qRT-PCR. ^a^ *P* < 0.05, versus the RTEC group; ^b^ *P* < 0.05, versus the HR RTEC group. **b** Expression of miR-223 in MSCs was detected by qRT-PCR. ^a^ *P* < 0.05, versus the normal cultured MSCs

**Fig. 3:**
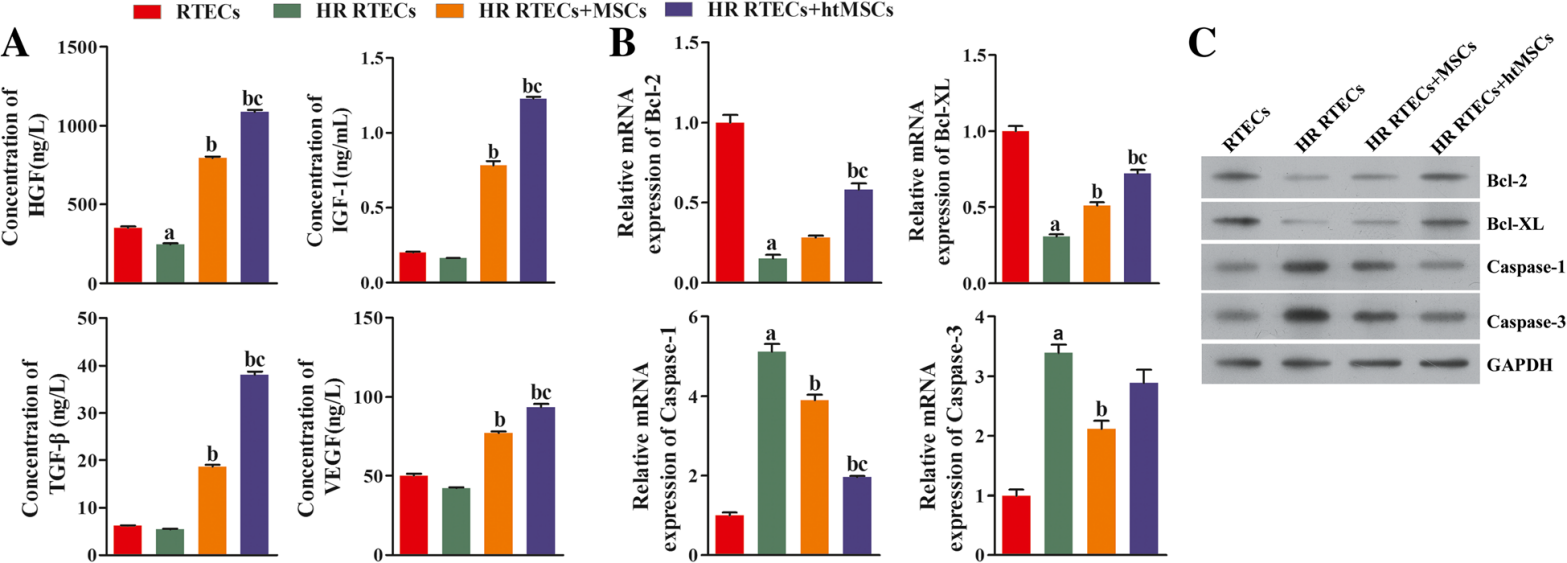
Expression of growth factors, chemokines, and antiapoptosis factors was enhanced by coculture with mesenchymal stem cells (*MSCs*), while expression of proapoptosis factors was suppressed. Renal tubular epithelial cells (*RTECs*) were treated with hypoxia/reoxygenation (*HR*) stimulation and then cocultured with MSCs or hypoxia-pretreated MSCs (*htMSCs*) in a double-chamber. Cells were harvested at 48 h. **a** The concentrations of hepatocyte growth factor (*HGF*), insulin-like growth factor-1 (*IGF-1*), transforming growth factor beta (*TGF-β*), and vascular endothelial growth factor (*VEGF*) in coculture supernatants were measured by ELISA. **b** Expression of B-cell lymphoma-2 (*Bcl-2*), B-cell lymphoma-XL (*Bcl-XL*), cysteine protease protein-1 (*caspase-1*), and cysteine protease protein-3 (*caspase-3*) in RTECs was detected by qRT-PCR. **c** Representative images of Western blot assays for apoptosis-related indicators. ^a^ *P* < 0.05, versus the RTEC group; ^b^ *P* < 0.05, versus the HR RTEC group; ^c^ *P* < 0.05, versus the HR RTEC + MSC group. *GAPDH* glyceraldehyde-3-phosphate dehydrogenase

The injuries induced by H/R decreased the miR-223 levels in RTECs (Fig. 2a). However, hypoxia treatment increased the levels of miR-223 expression in both MSCs (Fig. 2b) and RTECs (Fig. 2a). The levels of excreted protective cytokines HGF, IGF-1, TGF-β, and VEGF in the supernatants of coculture medium were significantly increased by treatment with MSCs when compared with those in the RTECs and HR RTECs groups (*P* < 0.05; Fig. 3a). Furthermore, the decreased expression levels of all the antiapoptosis indicators (including Bcl-2 and Bcl-XL) in the HR RTEC groups were restored after treatment with htMSCs (Fig. 3b and c), while the expression levels of proapoptosis indicators (including caspase-1 and caspase-3) were partially downregulated. The results obtained after administration of MSCs indicated that the MSCs had actively contributed to increasing the levels of miR-223 in H/R RTECs.

### Notch signaling in MSCs was initiated by hypoxia pretreatment

**Fig. 4:**
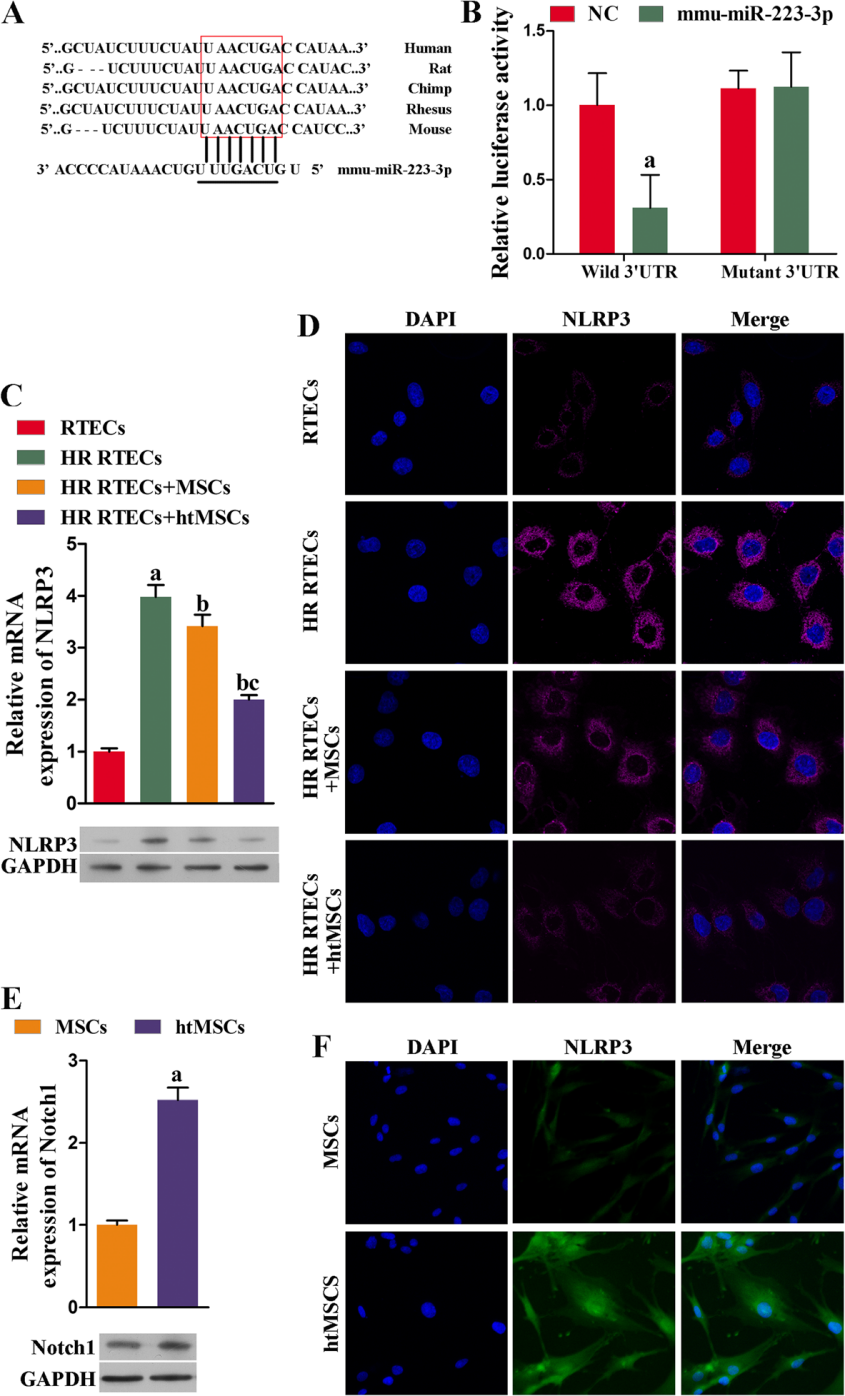
Expression of NLRP3 was downregulated in renal tubular epithelial cells (*RTECs*) cocultured with mesenchymal stem cells (*MSCs*), and Notch signaling in MSCs was initiated by hypoxia pretreatment. **a** The effect of miR-223 on NLRP3 expression. The binding site was between miR-223 and the 3′-UTR of NLRP3 mRNA. **b** Dual luciferase assay. *P < 0.05, versus the NC group. **c** RTECs were treated with hypoxia/reoxygenation (*HR*) stimulation and then cocultured with MSCs or hypoxia-pretreated MSCs (*htMSCs*) in a double-chamber. Cells were harvested at 48 h. Expression of NLRP3 in RTECs was detected by qRT-PCR. ^a^ *P* < 0.05, versus the RTEC group; ^b^ *P* < 0.05, versus the HR RTEC group; ^c^ *P* < 0.05, versus the HR RTEC + MSC group. **d** Representative images from the immunofluorescence assays used to detect NLRP3 in RTECs (×400 magnification). **e** Expression of Notch1 in MSCs was detected by qRT-PCR. ^a^ *P* < 0.05, versus the normal cultured MSCs. **f** Representative images from immunofluorescence assays used to detect Notch1 in MSCs (×400 magnification). *DAPI* 4,6-diamino-2-phenyl indole, *GAPDH* glyceraldehyde-3-phosphate dehydrogenase, *NC* negative control, *NLRP3* NLR family-pyrin domain containing 3, *UTR* untranslated region

The prosurvival mechanism of MSCs was further investigated at the molecular level. A bioinformatics analysis verified existence of a complementary binding site between miR-223 and the 3′-UTR of NLRP3 mRNA (Fig. 4a). A dual luciferase assay showed that treatment with the miR-223 mimic significantly decreased the relative amount of luciferase activity (Fig. 4b), indicating that miR-223 exerted a regulatory effect on NLRP3 mRNA. We also assessed the levels of NLRP3 expression in RTECs obtained from the different groups. NLRP3 expression in RTECs was first induced by H/R treatment and then suppressed in the cocultured groups (Fig. 4c and d), and this pattern was synchronized with corresponding changes in cell viability and apoptosis. The promoting role of NLRP3 in an acute renal injury was negatively regulated by miR-223. Because miR-223 secreted by MSCs is known to be transferred to RTECs via a paracrine mechanism, we investigated the levels of Notch1 expression in MSCs and found that Notch1 expression was significantly increased by hypoxia pretreatment (*P* < 0.05; Fig. 4e and f). This suggests that miR-223 in MSCs might be upregulated by Notch signaling.

### miR-223 contributed to the attenuation of H/R injuries in RTECs by suppressing NLRP3 expression

**Fig. 5:**
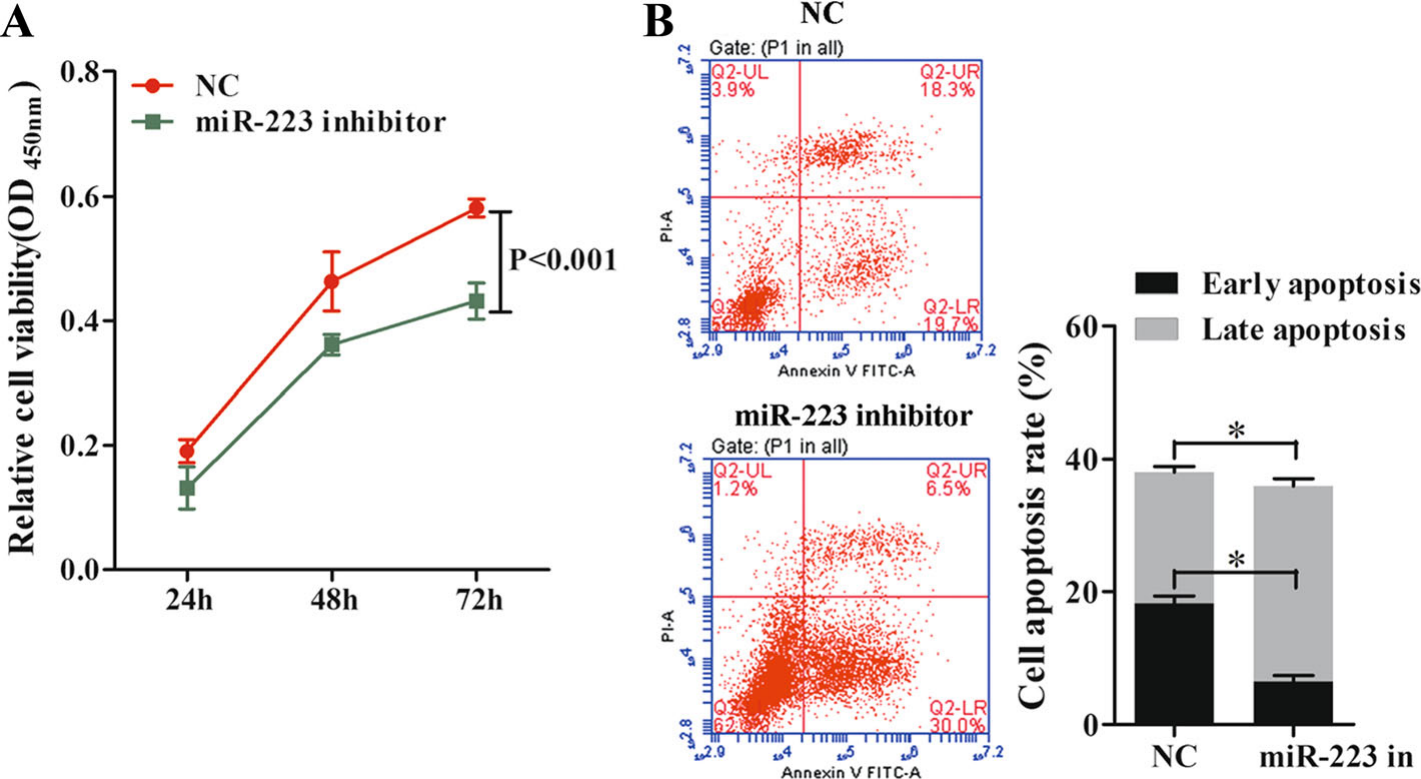
Downregulation of miR-223 in MSCs decreased cell viability and accelerated the apoptosis process in RTECs. All RTECs were treated with hypoxia/reoxygenation stimulation. The MSCs had been pretreated with a miR-223 inhibitor. The RTECs and MSCs were then cocultured in a double-chamber. **a** RTEC viability was measured with the CCK-8 assay. **b** Representative images of apoptotic RTECs at 48 h as detected by flow cytometry, *P < 0.05, *n* = 3. *NC* negative control, *OD* optical density, *PI* propidium iodide

**Fig. 6:**
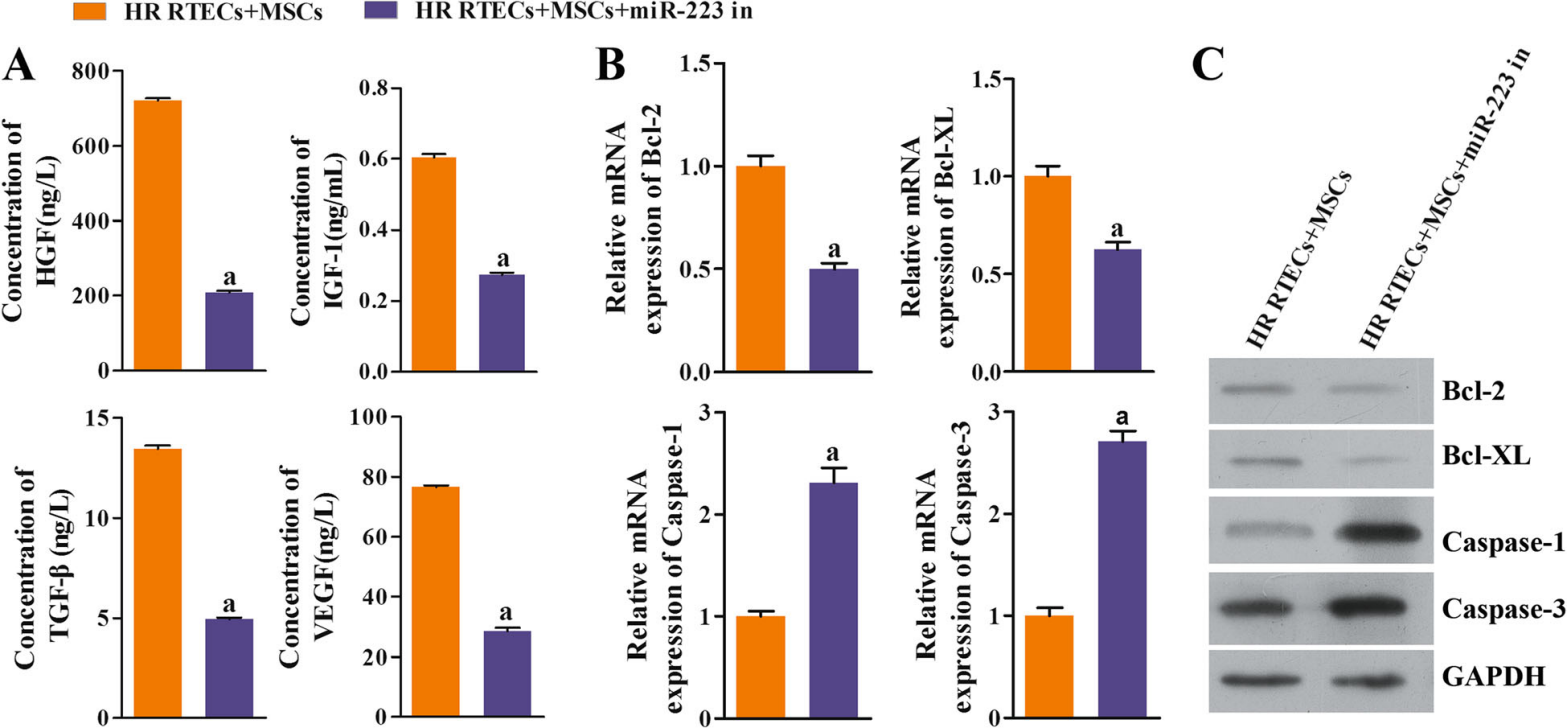
Expression of growth factors, chemokines, and antiapoptosis factors was suppressed by coculture with mesenchymal stem cells (*MSCs*) when miR-223 was inhibited; however, expression of proapoptosis factors was enhanced. All renal tubular epithelial cells (*RTECs*) were treated with hypoxia/reoxygenation (*HR*) stimulation. The MSCs had been transfected in advance with a miR-223 inhibitor (*miR-223 in*). The RTECs and MSCs were then cocultured in a double-chamber. **a** The concentrations of hepatocyte growth factor (*HGF*), insulin-like growth factor-1 (*IGF-1*), transforming growth factor beta (*TGF-β*), and vascular endothelial growth factor (*VEGF*) in coculture supernatants were measured with ELISA. **b** Expression of B-cell lymphoma-2 (*Bcl-2*), B-cell lymphoma-XL (*Bcl-XL*), cysteine protease protein-1 (*caspase-1*), and cysteine protease protein-3 (*caspase-3*) in RTECs was detected by qRT-PCR. **c** Representative images of Western blot assays for apoptosis-related indicators. ^a^ *P* < 0.05, versus the RTEC + MSC group. *GAPDH* glyceraldehyde-3-phosphate dehydrogenase

**Fig. 7:**
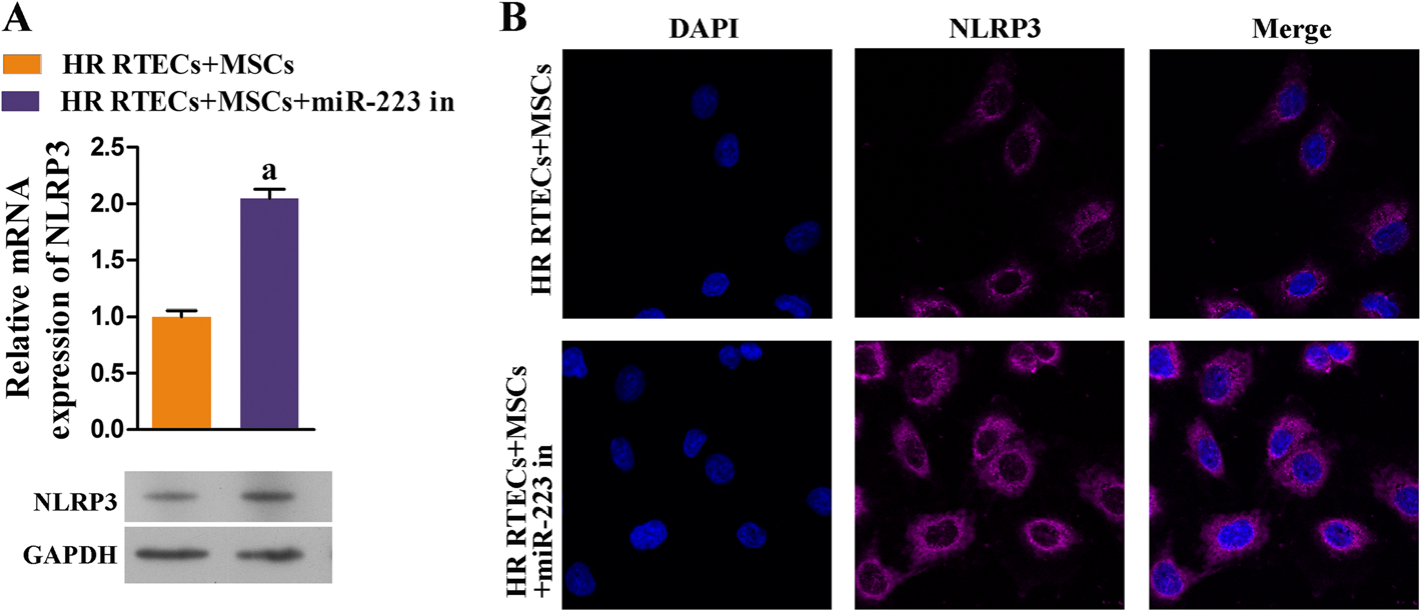
Expression of NLRP3 in renal tubular epithelial cells (*RTECs*) was upregulated after coculture with mesenchymal stem cells (*MSCs*), during which miR-223 was inhibited. All RTECs were treated with hypoxia/reoxygenation (*HR*) stimulation. The MSCs had been transfected in advance with a miR-223 inhibitor (*miR-223 in*). The RTECs and MSCs were then cocultured in a double-chamber. **a** Expression of NLR family-pyrin domain containing-3 (*NLRP3*) in RTECs was detected by qRT-PCR and Western blot assays. ^a^ *P* < 0.05, versus the RTEC + MSC group. **b** Representative images from the immunofluorescence assay for NLRP3 in RTECs. *DAPI* 4,6-diamino-2-phenyl indole, *GAPDH* glyceraldehyde-3-phosphate dehydrogenase

To verify that miR-223/NLRP3 signaling plays a key role in the kidney protective function of MSCs, we specifically inhibited miR-223 expression in MSCs. We found that the viability of cocultured RTECs became dramatically decreased concomitant with miR223 inhibition (*P* < 0.05; Fig. 5a), and the rates of RTEC apoptosis were accelerated (Fig. 5b). At the same time, the levels of cell protective cytokines (i.e., HGF, IGF-1, TGF-β, and VEGF) in the coculture supernatants declined (Fig. 6a). Moreover, the levels of antiapoptosis indicators also declined due to the knockdown of miR-223, while the expression levels of molecules known to promote apoptosis (i.e., caspase-1 and caspase-3) increased (Fig. 6b and c). The downregulation of miR-223 in MSCs induced NLRP3 expression, indicating that miR-223 plays a key role in the mechanism by which MSCs protect kidney tissue (Fig. 7a and b).

### In vivo implantation of MSCs ameliorated kidney I/R injuries via miR-223-mediated NLRP3 suppression

**Fig. 8:**
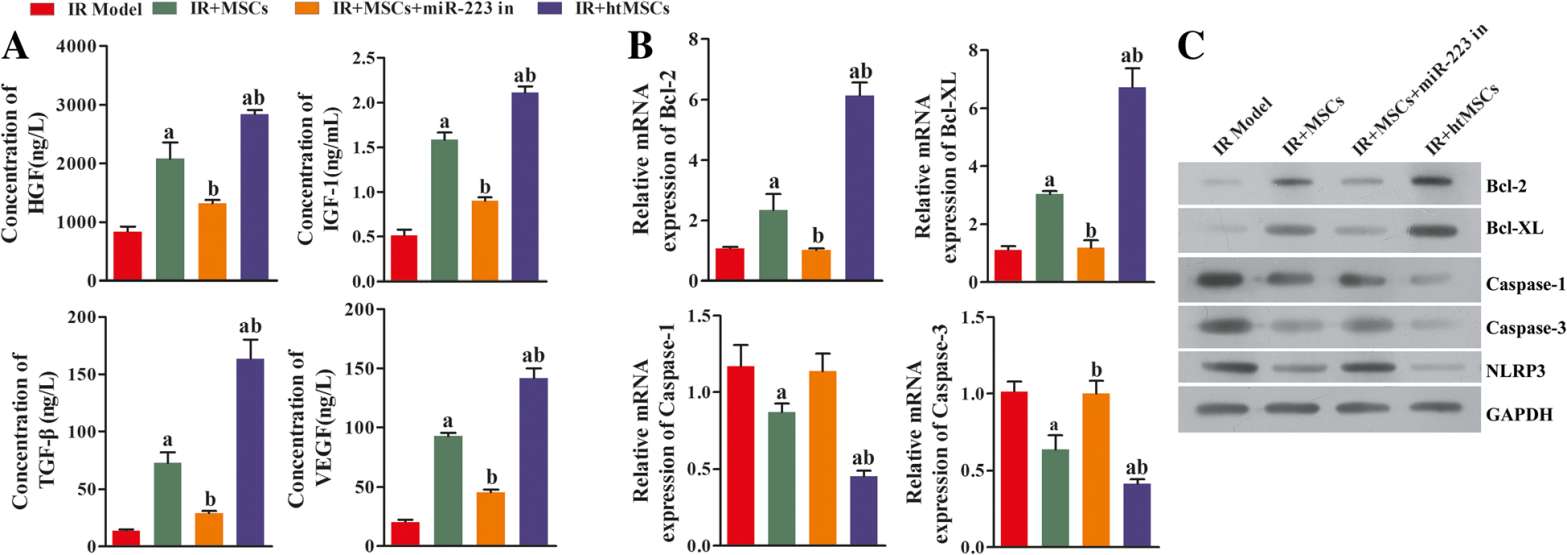
Knockdown of miR-223 blocked the protective effect of mesenchymal stem cells (*MSCs*) on renal tubular epithelial cells (*RTECs*). After the kidney ischemia/reperfusion (*IR*) KM/NIH mouse models were established, the mice were abdominally intravenously injected with MSCs, miR-223 inhibitor (*miR-223 in*) transfected MSCs or hypoxia-pretreated MSCs (*htMSCs*). Kidney tissues were harvested at 24 h after injection. **a** The concentrations of hepatocyte growth factor (*HGF*), insulin-like growth factor-1 (*IGF-1*), transforming growth factor beta (*TGF-β*), and vascular endothelial growth factor (*VEGF*) in blood samples were measured with ELISA. **b** Expression of B-cell lymphoma-2 (*Bcl-2*), B-cell lymphoma-XL (*Bcl-XL*), cysteine protease protein-1 (*caspase-1*) and cysteine protease protein-3 (*caspase-3*) in kidney tissue was detected by qRT-PCR. **c** Representative images from Western blot assays for apoptosis-related indicators. ^a^ *P* < 0.05, versus the IR model group; ^b^ *P* < 0.05, versus the I/R + MSC group. *GAPDH* glyceraldehyde-3-phosphate dehydrogenase, *NLRP3* NLR family-pyrin domain containing 3

**Fig. 9:**
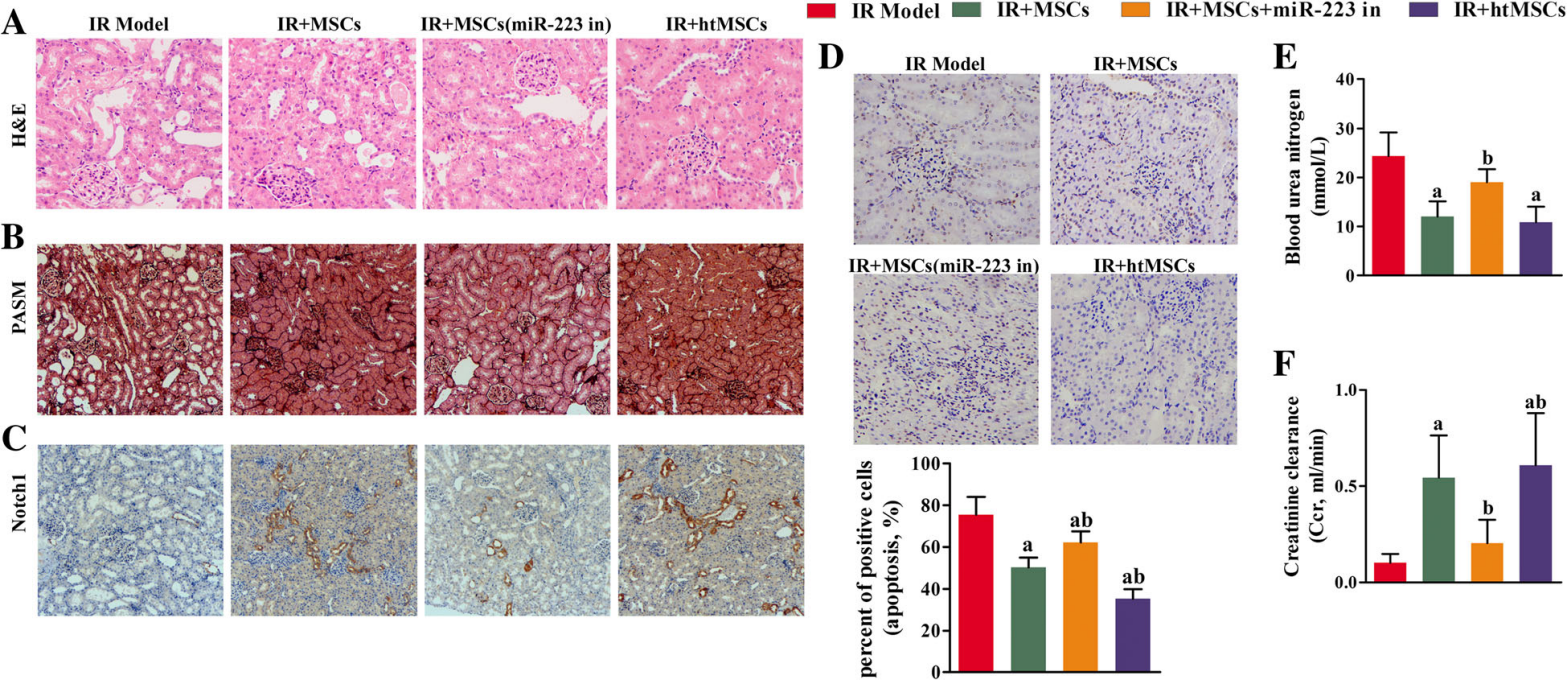
Perfusion of mesenchymal stem cells (*MSCs*) attenuated ischemia/reperfusion (*IR*)-induced damage to kidney tissue, and initiated Notch signaling in the mouse kidney. **a** Representative images of hematoxylin and eosin (*H&E*) stained tissue sections (×200). Administration of MSCs containing active miR-223 restored the integrity of cellular structures that had been damaged as a result of IR induction. **b** Representative images from periodic acid-silver methenamine (*PASM*) staining studies (×100 magnification). The collagen fibers are stained dark and the amount of kidney fibrosis was attenuated by perfusion with functional MSCs. **c** Representative images from immunochemical assays (×100 magnification); Notch1-positive cells are stained *brown*. Notch1 expression in the kidney cells of model mice was initiated by perfusion of MSCs transfected with a NC inhibitor or MSCs pretreated with hypoxia (*htMSCs*). **d** Representative images from TUNEL staining studies (×200 magnification). Apoptotic cells are stained *brown*, and their numbers were decreased after treatment with MSCs transfected with a negative control inhibitor or MSCs pretreated with hypoxia. Six fields were analyzed in the assay. **e** Blood urea nitrogen (BUN) assays showed that treatment with MSCs or htMSCs significantly decreased the levels of BUN compared to those in the IR group; however, a decrease in miR-223 expression reversed the reductions in BUN caused by MSCs or htMSCs. **f** The creatinine clearance rates were elevated in both the IR + MSC and IR + htMSC groups. The loss of miR-233 alleviated the increase in creatinine clearance caused by MSCs or htMSCs. ^a^ *P* < 0.05, versus the IR model group; ^b^ *P* < 0.05, versus the IR + MSC group. *miR-233 in* miR-233 inhibitor

To verify our findings in the H/R RTEC model, an in vivo renal I/R injury model was created using KM/NIH mice. Samples of kidney tissue from the treated mice were collected for RNA extraction. Next, qRT-PCR was performed to measure the mRNA levels of several cytokines. Mice injected with MSCs or htMSCs had increased levels of cell protective cytokines (HGF, IGF-1, TGF-β, and VEGF). The levels of those cytokines in mice injected with miR-223 knockdown MSCs were significantly lower than those in both the I/R + MSC and I/R + htMSC groups (*P* < 0.05). However, even though the miR-223 levels in MSCs had been reduced, the miR-233 levels in the I/R + MSC group were still higher than those in the I/R group (Fig. 8a). These results confirmed our findings from the in vitro assays, which showed that induction with I/R could initiate the innate production of miR-223. In accord with the results from in vitro assays, the expression levels of antiapoptosis molecules Bcl-2 and Bcl-XL were enhanced, while those of several proapoptosis molecules (caspase-1, caspase-3, and NLRP3) were suppressed in the groups treated with functional MSCs (Fig. 8b and c). Details of the histological changes seen in the mouse models are shown in Fig. 9. H&E staining revealed that administration of MSCs with active miR-223 restored cellular structures that had been damaged due to I/R induction (Fig. 9a). The degrees of kidney fibrosis were also reduced by administration of functional MSCs (Fig. 9b). Additionally, we also used an immunohistochemical assay to detect Notch1 expression in the RTECs obtained from our mouse model. Interestingly, both the expression and distribution of Notch1 in the mouse kidneys were increased (Notch1-positive cells were stained brown) (Fig. 9c), which might signify initiation of a self-protection response in I/R-injured kidney tissues. Our TUNEL staining results showed that the numbers of apoptotic RTECs were decreased after treatment with MSCs when compared with those numbers in the I/R group. This reduced level of apoptosis in the I/R + MSC group was absent when miR-223 was inhibited. When compared with the levels of apoptosis in the I/R and I/R + MSC groups, a significant inhibition of apoptosis was found after treatment with htMSCs (*P* < 0.05) (Fig. 9d). In a similar manner, results of BUN assays also indicated that treatment with MSCs or htMSCs significantly decreased the levels of BUN, whereas suppression of miR-223 impeded the reduction in BUN induced by MSCs or htMSCs (*P* < 0.05) (Fig. 9e). We also examined the creatinine clearance rates in mice which received the different treatments, and found that the creatinine clearance rates were significantly increased in the I/R + MSC group and were even upregulated in the I/R + htMSC group (Fig. 9f). Interestingly, downregulation of miR-223 significantly reduced creatinine clearance rates in the I/R + MSC group, indicating that miR-223 plays a role in the ability of MSCs to treat I/R-induced kidney injuries.

## Discussion

While therapies based on MSC transplantation have been successfully used to alleviate injuries in liver, heart, and renal models [13, 14, 24, 25], the underlying mechanisms involved in the protective effect of MSCs on injured tissues have never been fully understood. In the current study, we demonstrated that administration of MSCs could ameliorate kidney damage induced by I/R, and the related mechanism involved inhibition of NLRP3 activity via activation of miR-223.

MicroRNAs comprise a group of endogenous short noncoding RNAs which modulate the translation of targeted messenger RNAs by binding to the 3′-UTR of the targeted messenger RNA transcripts, thereby leading to RNA degradation and inhibition of protein synthesis [26]. In recent years, numerous associations between renal I/R and specific miRNAs have been identified. Wei et al. [27] found that deletion of *Dicer* in the proximal tubular epithelium protected kidney tissue from I/R-induced injuries, and this effect was correlated with changes in the expression of specific miRNAs [27]. Moreover, in a unilateral warm renal ischemia model created in mice, nine miRNAs were found to be differentially expressed in the ischemic kidneys when compared with the control kidneys [28]. Our previous studies showed that the protective effect of MSCs on a DCD rat kidney was associated with activation of miR-223, as well as the specific suppression of its targeted gene, *NLRP3*. Therefore, it was reasonable to hypothesize that miR-223 plays a key role in I/R-induced injuries to the kidney, as well as the ability of MSCs to protect kidney tissue. For the following reasons, this hypothesis was validated in the current study: 1) RTECs from an induced H/R model displayed significantly decreased levels of miR-223 expression in vitro; and 2) knockdown of miR-223 in MSCs impaired the treatment effect of MSCs on RTECs in vitro, and on renal I/R mice in vivo. A previous study reported that MSCs secrete exosomes capable of transporting miR-223 into nearby cells for the purpose of regulating targeted pathways [24]. In the present study, results obtained after the coculture of MSCs with RTECs without physical contact partially confirmed that idea. After administration of MSCs alone, the levels of miR-223 in RTECs dramatically increased. Moreover, miR-223 appeared to affect the amounts of growth factors and chemokines secreted by MSCs. It was noteworthy that suppression of miR-223 decreased HGF, IGF-1, TGF-β, and VEGF production *both* in vitro and in vivo. Because secretion of these polypeptides contributes to the ability of MSCs to help increase epithelial proliferation and modulate inflammation or angiogenesis, those polypeptides can be considered as therapeutic candidates for treating renal injuries [29–31]. Taken together, the central role played by miR-223 during the onset of a renal I/R injury was affirmed. Thus, the upregulation of miR-223 in kidney tissues by therapeutic modalities such as MSCs is a promising method for treating renal failure induced by an I/R process.

Our current study investigated the mechanism by which miR-223 functions as a key mediator between MSCs and RTECs. Previous studies have shown that Notch1 receptors are expressed in MSCs, which were proven to serve as upstream regulators of miR-223 [21, 22]. Thus we investigated the relationship between Notch1 expression and miR-223 levels in MSCs. As it has been demonstrated that hypoxia-treated MSCs can initiate Notch signaling [32], we found that the levels of miR-223 expression in MSCs were significantly increased. Those results proved the positive correlation between miR-223 expression and activation of Notch1.

Thereafter, we investigated the levels of miR-223 target gene (*NLRP3*) expression in both RTECs and kidney tissue [20, 33]. Nod-like receptors are crucial signaling regulators involved in the formation of intracellular macromolecular complexes, and contribute to host immune responses [34]. Shigeoka et al. [19] showed that NLRP3 promoted tissue injury by exerting a direct effect on the renal tubular epithelium in an inflammasome-independent manner [19]. Our study showed that upregulated levels of NLRP3 were closely associated with induction of an I/R injury both in vitro and in vivo. At the molecular level, enhanced NLRP3 activity contributed to the onset of inflammation by increasing caspase-1 levels [35], and to the initiation of mitochondrial pathway-mediated apoptosis by increasing caspase-3 levels and decreasing Bcl-2 and Bcl-XL levels [36]. Administration of MSCs suppressed the function of NLRP3 both in RTECs and kidney tissues, whereas knockdown of miR-223 blocked the effect of MSCs. These findings indicate that MSCs protect RTECs and kidney tissue by inhibiting NLRP3 via miR-223, and this effect was even more evident when Notch signaling in MSCs was activated by hypoxia treatment. Additionally, during the immunochemical detection of Notch1 in mouse kidney tissues, it was found that Notch1 levels in the MSCs of I/R-treated mice increased in synchrony with upregulation of miR-223 in the corresponding samples. This finding suggests activation of a self-protective signaling pathway in I/R-injured kidneys. Data from apoptosis, BUN, and creatinine clearance studies also suggested a significant role for miR-223 in the alleviation of kidney I/R injuries by implanted MSCs. However, a determination of whether Notch1 activation in kidney tissue represents a feedback response to miR-223 or occurs due to direct contact with MSCs in vivo requires further investigation.

## Conclusions

Our findings demonstrate that MSCs ameliorated I/R-induced renal failure via a paracrine mechanism. The exosomal miR-223 secreted by MSCs was transferred into RTECs and attenuated damage to renal cells after NLRP signaling was suppressed. Moreover, the protective effect of MSCs on kidney tissue was enhanced by hypoxia pretreatment, which increased miR-223 levels by activating Notch1 expression. Our studies with an in vivo mouse model show that the therapeutic effect of MSCs on I/R-induced renal injuries was reduced by suppression of the miR-223/Notch signaling pathway. When taken together, our data show that MSCs can attenuate renal I/R-induced injuries by transporting miR-223 into kidney cells and inhibiting NLRP3.

## Abbreviations

Bcl-2: B cell leukemia/lymphoma 2
Bcl-XL: B cell leukemia/lymphoma 2 -like 1
BUN: Blood urea nitrogen
Caspase-1/3: Cysteinyl aspartate specific proteinase-1/3
DAPI: 4,6-Diamino-2-phenyl indole
DMEM: Dulbecco’s modified Eagle’s medium
ELISA: Enzyme-linked immunosorbent assay
FBS: Fetal bovine serum
GAPDH: Glyceraldehyde-3-phosphate dehydrogenase
H&E: Hematoxylin and eosin
H/R: Hypoxia/reoxygenation
HGF: Hepatocyte growth factor
htMSC: Hypoxia-pretreated mesenchymal stem cell
I/R: Ischemia/reperfusion
IGF-1: Insulin-like growth factor-1
MSC: Mesenchymal stem cell
NLRP3: NLR family-pyrin domain containing 3
PASM: Periodic acid-silver methenamine
PBS: Phosphate-buffered saline
qRT-PCR: Quantitative real-time polymerase chain reaction
RTEC: Renal tubular epithelial cell
TGF-β: Transforming growth factor beta
TUNEL: Terminal deoxynucleotidyl transferase-mediated nick-end labeling
UTR: Untranslated region
VEGF: Vascular endothelial growth factor
